# The development of integrated diabetes care in the Netherlands: a multiplayer self-assessment analysis

**DOI:** 10.1186/s12913-017-2167-6

**Published:** 2017-03-21

**Authors:** Nick Zonneveld, Lidewij E. Vat, Hans Vlek, Mirella M. N. Minkman

**Affiliations:** 1 Vilans, National Center of Excellence in Long-term Care, Catharijnesingel 47, PO Box 8228, 3503 RE Utrecht, The Netherlands; 2 Memorial University Newfoundland, St. John’s, Canada; 3 University of Tilburg/TIAS, Tilburg, The Netherlands

**Keywords:** Diabetes, Integrated care, Care groups, Care networks, Development, Quality management, Stakeholder groups

## Abstract

**Background:**

Since recent years Dutch diabetes care has increasingly focused on improving the quality of care by introducing the concept of care groups (in Dutch: ‘zorggroepen’), care pathways and improving cooperation with involved care professionals and patients. This study examined how participating actors in care groups assess the development of their diabetes services and the differences and similarities between different stakeholder groups.

**Methods:**

A self-evaluation study was performed within 36 diabetes care groups in the Netherlands. A web-based self-assessment instrument, based on the Development Model for Integrated Care (DMIC), was used to collect data among stakeholders of each care group. The DMIC defines nine clusters of integrated care and four phases of development. Statistical analysis was used to analyze the data.

**Results:**

Respondents indicated that the diabetes care groups work together in well-organized multidisciplinary teams and there is clarity about one another’s expertise, roles and tasks. The care groups can still develop on elements related to the management and monitoring of performance, quality of care and patient-centeredness. The results show differences (*p < 0.01)* between three stakeholders groups in how they assess their integrated care services; (1) core players, (2) managers/directors/coordinators and (3) players at a distance. Managers, directors and coordinators assessed more implemented integrated care activities than the other two stakeholder groups. This stakeholder group also placed their care groups in a further phase of development. Players at a distance assessed significantly less present elements and assessed their care group as less developed.

**Conclusions:**

The results show a significant difference between stakeholder groups in the assessment of diabetes care practices. This reflects that the professional disciplines and the roles of stakeholders influence the way they asses the development of their integrated care setting, or that certain stakeholder groups could be less involved or informed.

**Electronic supplementary material:**

The online version of this article (doi:10.1186/s12913-017-2167-6) contains supplementary material, which is available to authorized users.

## Background

Since decades the number of people suffering from diabetes worldwide is growing [1]. According to the Netherlands National Institute for Public Health and the Environment (RIVM), more than 600,000 people were suffering from diabetes in the Netherlands in 2003 [2]. In 2011 approximately 830,000 people suffered from diabetes [3]. It is expected that by 2025, about 1.2 million people will suffer from diabetes in the Netherlands in 2025 [4] on a population of approximately 17 million people. In 2011 the total costs of diabetes care in the Netherlands amounted to almost EUR 1.7 billion (1.9% of the total healthcare costs in the Netherlands) [5].

In the Netherlands, like in other western countries, diabetes disease management programmes have been introduced in primary care settings as a response to the growing health care costs and demand for quality improvement in diabetes care [6]. In general, disease management refers to a patient-centered approach that aims to improve, structure and coordinate delivery of health care services to a specific patient group [7]. In 2013, more than 80% percent of diabetes care in the Netherlands was delivered in primary care settings [8], including former secondary care such as delivering insulin therapy [9]. In practice, general practitioners delegated most of diabetes care activities to practice nurses or specially trained diabetes nurses [10].

Since 2007, the Dutch Ministry of Health, Welfare and Sport, encourages integration and cooperation between involved professionals in diabetes healthcare in the Netherlands. In 2009, the Dutch Organization for Health Research and Development (ZonMw) started a national disease management program to strengthen disease management in the Netherlands [11]. In 2010, a new finance structure for chronic care, called bundled payments, was introduced. By the introduction of bundled payments [12] a new organizational construct was introduced: the care group (in Dutch: ‘zorggroep’). A care group is “an organization in which care providers are associated who are responsible for the delivering of chronic care to a specific patient population in which a bundled payment contract is used” [13]. In the Netherlands more than a 100 care groups are active nowadays. In these care groups general practitioners, diabetes nurses, dieticians, internists and many more professionals involved in delivering integrated diabetes care are contracted [14].

To study the current situation and development of integrated diabetes care in the Netherlands, a benchmark study was carried out. The individual results of each care group and total benchmark results were presented to each care group. The total results were presented in a benchmark report [15]. The aim of this study was to compare the development of integrated diabetes care provided by different care groups, to get a deeper understanding of the development of integrated diabetes care in the Netherlands and to make recommendations for practice. Next to delivering an overview of the state of the art, a special interest was in the analyses of the assessment of different involved stakeholders.

## Methods

### Development model for integrated care

**Fig. 1:**
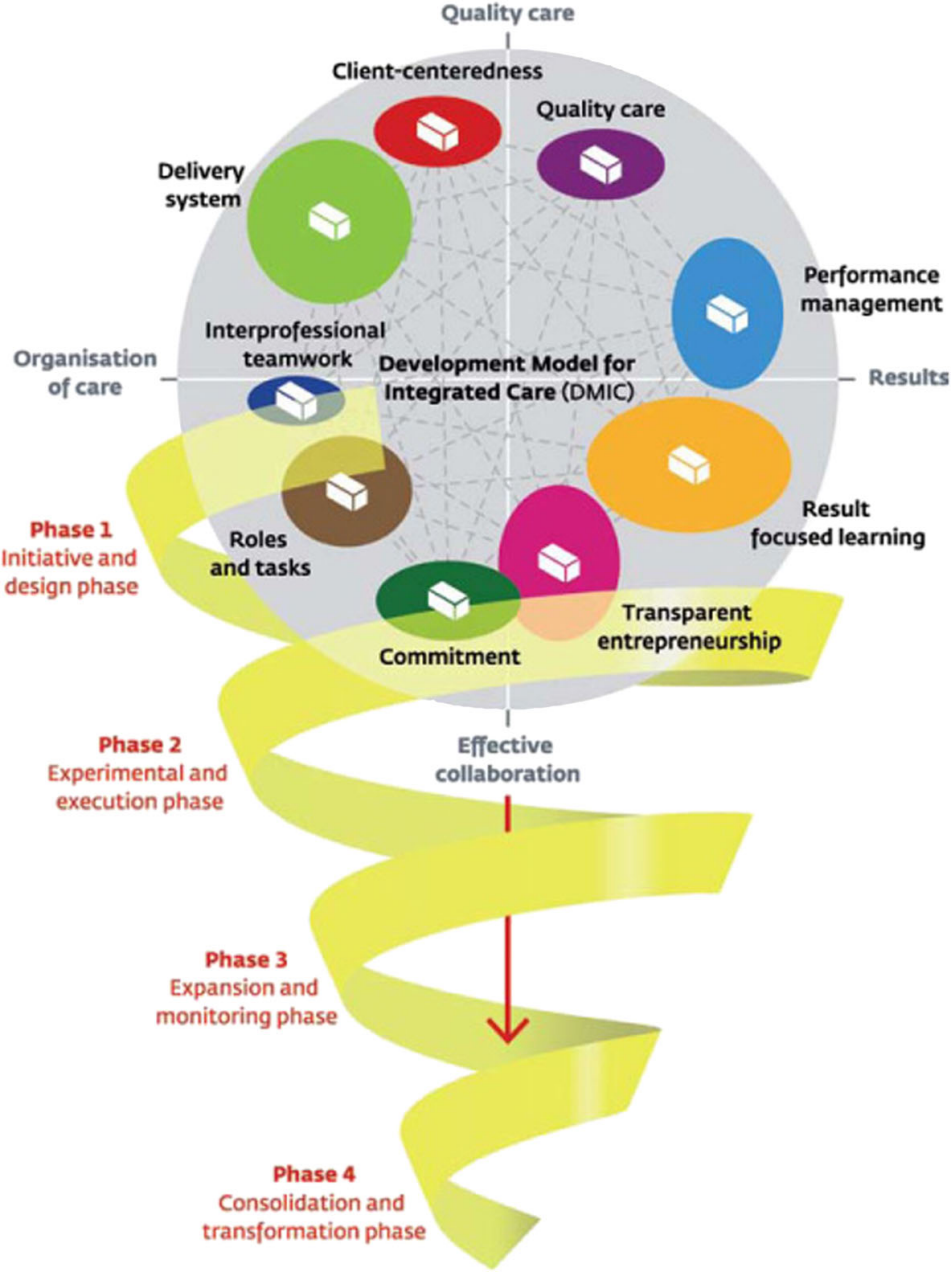
The Development Model of Integrated Care – Copy of the original figure [16]

To integrate care from multiple providers into a coherent and streamlined client-focused service, a large number of activities and agreements have to be implemented. Streamlining information flows and adequate transfers of clients are such examples. Due to the large range of possible activities, it is proven to be difficult in practice to identify essential activities and to determine with which ones to start and implement in what order. The Development Model for Integrated Care (DMIC, see Fig. 1) describes the important clusters (groups of activities) and elements (activities) for developing and implementing coherent and seamless care in integrated care services [16].

The DMIC is a systematically developed model, based on a literature study, a Delphi study and multiple questionnaire researches. The DMIC is validated in stroke, acute myocardial infarct (AMI) and dementia services in the Netherlands and in Canada [16–19]. Additionally, a pilot study in a Dutch diabetes service showed that the model is suitable for a diabetes care setting as well (relevance scores of all clusters >85% [20]). Eventually 89 relevant elements of integrated care were determined, which are grouped into nine clusters.

The nine key clusters of the DMIC are:

1. Patient centeredness (9 elements), for example ‘Collaboratively offering client information of the care partners’.
2. Delivery system (18 elements), for example ‘Using a single client-monitoring record accessible to all care partners’.
3. Performance management (16 elements), for example ‘Defining performance indicators to evaluate the results of the integrated care delivered’.
4. Quality care (5 elements), for example ‘Systematically assessing the needs of the clients in the care chain’.
5. Result-focused learning (12 elements), for example ‘Stimulating a learning culture and continuous improvement in the care chain’.
6. Inter-professional teamwork (3 elements), for example ‘Working in multidisciplinary teams’.
7. Roles and tasks (8 elements), for example ‘Reaching agreements among care partners on tasks, responsibilities and authorizations’.
8. Commitment (11 elements), for example ‘Signing collaboration agreements among the care partners’.
9. Transparent entrepreneurship (7 elements), for example ‘Making a commitment to a joint responsibility for the final goals and results to be achieved’.

Furthermore, the model offers a distinction of four phases in integrated care development:

1. The initiative and design phase. Key words: Exploring possibilities/impossibilities, ambitions and chances, (project) design and collaboration agreements.
2. The experimental and execution phase. Key words: Writing down aims and content of the collaboration, coordination at care chain level, experimenting and reflecting.
3. The expansion and monitoring phase. Key words: Further development and maturity, monitoring and improving results, new questions and innovation.
4. The consolidation and transformation phase. Key words: Continuous improvement, new ambitions, structures fitting the integrated care programme (organizational structures, integral financing).

The 89 elements are related to the four development phases. Each phase consists of a top-10 of most important elements in that phase of development [18, 21].

According to the DMIC, the more elements of the development model for integrated care are present in a service, the more developed the service is. This study focuses on the identification of these elements in Dutch diabetes care.

The Development Model of Integrated Care – Copy of the original figure [16].

### Self-assessment web based questionnaire

To assess the 36 diabetes networks, a digital web-based self-assessment questionnaire was used (see Additional file 1 for the English copy of the questionnaire). The questionnaire is completely based on the validated DMIC [16] and consists of three main parts:

- *Part A: General information about the care group* The year of the start of the cooperation collaboration, involved partners and coordination of the care group.
- *Part B: Relevance and presence of the elements per cluster* In this part of the questionnaire, the 89 elements of the nine clusters of the DMIC are shown per cluster. First, the respondents indicated the relevance per element. If relevant, they rated whether this element was present in the care group or not, or if no judgement could be made. When respondents indicated that an element is not present (yet), they indicated whether this element has priority to be implemented or not.
- *Part C: Phases of development* This section is about the four development phases. First, a description of each phase was given. Then, the respondents indicated which phase described the current situation in their care group the best. At last, the respondents were asked to explain their answers by for instance mentioning facilitators for development.

#### Stakeholder groups

For answering the last research question about the different stakeholder groups in the care groups, three actor groups were distinguished:

1. Core players who have the most regular contact with the diabetes patients: general practitioners, dieticians and practice/diabetes nurses.
2. Managers/directors/coordinators: all managers, directors and coordinators.
3. Players at a distance: all other health professionals, as optometrists, podiatrists and internists.

### Respondents, respondent selection and analysis

The web-based questionnaire was sent out to 36 care groups in the Netherlands that were contracted by a Dutch large health care insurer. The coordinators of the care groups selected the respondents. Each care group invited eight to twelve partners who are closely involved in the local integrated diabetes care network. To include similar representatives per care group the advice was to include at least two general practitioners, one practice nurse or diabetes nurse, one dietician, one ophthalmologist or optometrist, one podiatrist, one internist and one project coordinator/director. If appropriate, respondents from other disciplines could also participate if they were closely involved in the care group and involved in diabetes care. Some care groups did invite a respondent who was less involved, to get an idea of how they look at the collaboration. These were for example a physiotherapist and a pharmacist.

Out of the 335 respondents of 36 care groups, 275 filled out the self-assessment completely (response rate 82%). Reasons for non-response (*n* = 37) were merely holidays and a lack of time for participation. A number of 23 respondents did not complete the questionnaire. The data of seven respondents who were not able to complete the questionnaire with sufficient data (>70% of the elements answered as ‘I cannot judge that’/‘not relevant’) has been left out the analysis. In total 268 questionnaires were included in the analysis. In Table 1 the professional disciplines of the respondents are shown, categorized in stakeholder groups.

**Table 1:** Professional disciplines of the respondents, categorized in stakeholder groups (*n* = 268)

Stakeholder group	Professional discipline	*n*
Core players (*n* = 159):	General practitioner	67
	Practice nurse/Nurse	53
	Dietician	39
Director/Manager/Coordinator (*n* = 36):	Director/Manager/Coordinator	36
Players at a distance (*n* = 73):	Internist	23
	Pedicure/Podiatrist	23
	GPs laboratory	7
	Ophthalmologist/Optometrist	7
	Pharmacist	5
	Physiotherapist	3
	Patient representative	1
	Other health professionals	4
	Total	268

Descriptive, ANOVA variance, Pearson Chi-Square, Phi and Cramér’s V analyses were used to analyze the results. Based on their results, every care group received a report with the results and suggestions for further development of their diabetes care service. With each care group a feedback session was organized, to present and discuss the results. Each participating care group received three to five recommendations for further development, which were open for discussion.

## Results

The number of respondents of the questionnaire varied with on average seven to eight (7.4) respondents per care group, with a minimum of five and a maximum of twelve respondents per care group. There was no difference between larger or bigger care groups regarding the number of respondents. Table 2 gives an overview of the number of respondents per care group.

**Table 2:** Number of respondents per care group (*n* = 268)

Respondents (*n)*	Care groups (*n)*
5 respondents	3
6 respondents	10
7 respondents	10
8 respondents	2
9 respondents	7
10 respondents	1
11 respondents	2
12 respondents	1
Total	36

### Relevance

Per element the respondents of the care groups indicated whether they found the element is or is not relevant for their diabetes care practice. The relevance scores per cluster were all higher than 90% and varied between 91% (cluster 2, ‘Delivery system’) and 97.3% (cluster 7, ‘Roles and tasks’). Table 3 shows the relevance scores of all clusters. Every cluster contains a number of elements (range 3 to 18). On the basis of the relevance score per element, the average scores per cluster were calculated. A score of 100% in a cluster means that all respondents indicated that all elements of that cluster were relevant for their diabetes care.

**Table 3:** Relevance scores per cluster of the DMIC [16] (*n* = 268)

Cluster	Relevance score *(%)*
(1) Patient centeredness (9 elements)	95.4
(2) Delivery system (18 elements)	91.0
(3) Performance management (16 elements)	96.7
(4) Quality care (5 elements)	92.7
(5) Result-focused learning (12 elements)	92.9
(6) Inter-professional teamwork (3 elements)	97.1
(7) Roles and tasks (8 elements)	97.3
(8) Commitment (11 elements)	93.6
(9) Transparent entrepreneurship (7 elements)	93.8

### Descriptive results on care group level

**Fig. 2:**
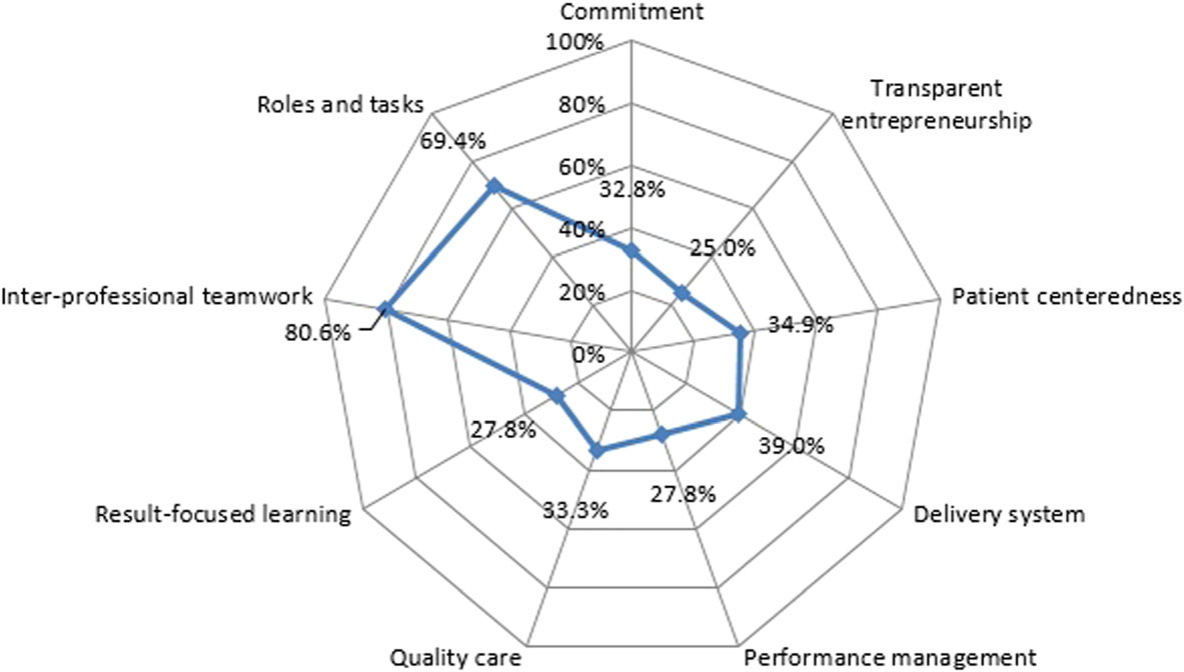
Self-assessment presence scores of per care group per cluster (*n* = 36)

Figure 2 gives an overview of the implemented integrated care elements in the 36 care groups on care group level, grouped into the nine clusters. The blue line in the graph represents the average percentage of the present elements per cluster. An element is considered to be present, when at least 70% of the respondents in a care group indicates that the element is present. The closer the blue line is to the outer ring, the higher the average percentage of present DMIC elements in the care groups is.

On average, the respondents of the care groups evaluated their care groups with the highest scores of present elements on the clusters ‘Inter-professional teamwork’ (80.6%) and ‘Roles and tasks’ (69.4%). The scores on the clusters ‘Delivery system’ (39.0%), ‘Patient centeredness’ (34.9%), ‘Quality care’ (33.3%), ‘Commitment’ (32.8%), ‘Performance management’ (27.8%), ‘Result-focused learning’ (27.8%) and ‘Transparent entrepreneurship’ (25.0%) are lower.

### Analysis on stakeholder groups

Table 4 shows the differences on average cluster scores between the three stakeholder groups. To examine the relationships between self-assessment scores per cluster and the stakeholder groups, one-way ANOVA analyses [22] were conducted.

**Table 4:** Mean (Standard Deviation) self-assessment presence scores per cluster per stakeholder group, including one-way ANOVA results (*n* = 268)

	Core players (*n* = 159)	Managers/directors/coordinators (*n* = 36)	Players at a distance (*n* = 73)	Total (*n* = 268)		
Cluster	*M (SD)*	*M (SD)*	*M (SD)*	*M (SD)*	*Fisher*	*p-value*
(1) Patient centeredness (9 elements)	5.07 (2.09)	6.03 (1.67)	3.52 (2.13)	4.78 (2.21)	22.03	<0.01
(2) Delivery system (18 elements)	10.1 (3.89)	11.2 (2.34)	7.73 (4.22)	9.60 (4.00)	13.35	<0.01
(3) Performance management (16 elements)	8.37 (4.03)	11.3 (2.41)	5.29 (4.41)	7.92 (4.38)	29.90	<0.01
(4) Quality care (5 elements)	2.13 (1.05)	2.6 (1.01)	1.85 (1.08)	2.12 (1.08)	7.24	<0.01
(5) Result-focused learning (12 elements)	6.05 (3.31)	8.92 (2.06)	4.23 (3.61)	5.94 (3.54)	25.10	<0.01
(6) Inter-professional teamwork (3 elements)	2.42 (0.84)	2.61 (0.60)	2.01 (1.07)	2.33 (0.91)	7.19	<0.01
(7) Roles and tasks (8 elements)	5.87 (2.02)	6.44 (1.52)	5.00 (2.56)	5.71 (2.17)	6.65	<0.01
(8) Commitment (11 elements)	6.12 (3.07)	7.69 (2.45)	4.40 (3.39)	5.86 (3.24)	15.13	<0.01
(9) Transparent entrepreneurship (7 elements)	3.89 (2.04)	5.06 (1.62)	2.25 (2.26)	3.60 (2.24)	26.50	<0.01
Total DMIC (89 elements)	50.0 (17.2)	61.9 (10.8)	36.3 (20.4)	47.9 (19.2)	28.88	<0.01

There are significant differences between the stakeholder groups in each cluster and in the total DMIC score. In each cluster the managers/directors/coordinators stakeholder group meanly assessed the most elements as present, followed by the core players and the players at a distance.

Table 5 shows the differences on phase estimation between the stakeholder groups. To examine the relationship between phase estimation and stakeholder group, a contingency table has been made and Pearson Chi-Square, Phi and Cramér’s V tests were conducted [22].

**Table 5:** Phase estimation scores (%) per stakeholder group, including Pearson Chi-Square, Phi and Cramér’s V results

	Core players (*n* = 159)	Managers/directors/coordinators (*n* = 36)	Players at a distance (*n* = 73)	Total (*n* = 268)
Phase:	*n (%)*	*n (%)*	*n (%)*	*n (%)*
(1) Initiative and design phase	11 (6.90)	-	8 (11.0)	19 (7.10)
(2) Experimental and execution phase	24 (15.1)	4 (11.1)	32 (43.8)	60 (20.4)
(3) Expansion and monitoring phase	73 (45.9)	18 (50.0)	23 (31.5)	114 (42.5)
(4) Consolidation and transformation phase	51 (32.1)	14 (38.9)	10 (13.7)	75 (28.0)

*χ* ^2^ = 35.712, *df* = 6, *p* < 0.0001

Φ = .365, *p* < 0.0001

*V* = .258, *p* < 0.0001

Concerning the four phases of the DMIC, there are significant differences between the stakeholder groups as well. The respondents of the managers/directors/coordinators stakeholder group rank their care group in a further phase of development than respondents of the core players stakeholder group, followed by the respondents of the players at a distance stakeholder group.

For example, 43,8% of the players at a distance rank the development of their care group as in the experimental and execution phase (phase 2), against respectively 15,1 and 11,1% of the core players and managers/directors/coordinators. The opposite can be seen in the scores concerning the consolidation and transformation phase (phase 4). Respectively 32,1 and 38,9% of the core player and manager/director/coordinator stakeholder groups rate their care group as in phase 4, against 13,7% of the players at a distance stakeholder group.

## Discussion

### Study findings and reflections

This study presents the results of a self-assessment monitoring of 36 diabetes care groups in the Netherlands. The self-assessment had a response rate of 82% and the respondents indicated the elements of all nine clusters with high relevance scores. The results show that care groups developed a disease management program with clear roles and tasks. The clusters ‘Inter-professional teamwork’ and ‘Roles and tasks’ are assessed with high scores. Professionals work together in multi-disciplinary teams according to evidence-based guidelines and standards, with clarity about another’s expertise, roles and tasks. From a patient perspective, these are essential elements. They help patients to find their way in the healthcare system and ensure coordinated care. Results show also area for improvement on clusters as ‘Performance management’, ‘Quality care’ and ‘Patient centeredness’. A recently published Dutch study on 23 diabetes care groups showed the same picture: the care groups mostly paid attention to aspects related to organization and management and less to aspects related to patient-centeredness and quality improvement [23]. It seems that care groups have been focused on who does what, for example referring tasks from general practitioners to diabetes nurses and less attention has been given on aspects related to performance management. Often collaboration requires clear agreements about roles and tasks before performances can be management.

Secondly, there is a significant relationship between the self-assessment scores and the stakeholder groups. The stakeholder groups significantly identify different numbers of present activities in their diabetes care practices. These differences are visible on all nine clusters and on the total assessment of all elements of the DMIC. On each cluster the manager/directors/coordinators group assess the most present elements, followed by the core player group and at last the players at a distance. The same applies to the development phase estimation of the respondents. On average, managers, directors and coordinators indicate that their diabetes care practices are in a further development phase than the respondents of the two other stakeholder groups do. In comparison with the other stakeholder groups, players at a distance assess their diabetes care practices significantly less developed.

The differences between the stakeholder groups have been discussed in the feedback conversations with the stakeholders and with the total group. An explanation could be that the players at a distance are less involved with the developmental and implementation processes of the care groups. Another explanation could be that the players at a distance are less informed about new activities and steps to further develop the diabetes care practice. The managers, coordinators and directors usually are the initiators of the care groups or the persons that are responsible to manage and develop the integrated diabetes care. Most of the key players are actually employed in the care groups and they are working daily on the further refinement of the care group and the implementation of integrated diabetes care. So, the players at a distance might have less contact with their care group colleagues than the other stakeholder groups. Through this they might be less involved and informed. Analyses in a recently published Canadian study [19], which used the DMIC as a framework for studying integrated services networks in Quebec, showed differences between stakeholder groups as well. They found that “nurses in management roles identified on average a significantly higher percentage of present activities than those with more clinical functions”. The results of these studies suggest that the professional background and the role a person takes in their integrated care service influences the way a person assesses the development of integrated care. From a patient’s perspective, this is worrying. The different views of the stakeholders could have an impact on the quality of care for patients.

### Study limitations and implications

This study has some limitations. Some respondents indicated that they found the survey too extensive and/or difficult. Especially players at a distance found some questions difficult. However, the observation that some players at a distance found some questions difficult might be also seen a result of the self-assessment. An explanation could be that these respondents are less informed about the development of the diabetes care group or have less interest in the organizational aspects of diabetes care. Also the number of respondents in the three stakeholder groups core players (*n* = 159), managers/directors/coordinators (*n* = 36) and players at a distance (*n* = 73) varied. However, this represents how the diabetes care groups are organized in practice in the Netherlands. Another important thing to keep in mind is that the DMIC focuses on activities that can be undertaken that contribute to integrated care. It does not focus on outcomes. Therefore, no conclusions can be drawn about the outcomes of the delivered care, such as health, quality of life, costs or efficiency. This is an interesting suggestion for further research.

### Practical implications and further research

Several recommendations for practice could be made. First of all, a shift in focus from roles and tasks to a joint responsibility for the final goals and results is recommended for further development of diabetes care. Care groups could develop more by improving on aspects related to performance management, quality management and patient-centeredness, as also mentioned in other Dutch studies [23, 24]. Additionally, many care groups could develop more by organizing multidisciplinary result focused learning in their working practices, for instance by giving each other feedback on a structural basis. Analyses of errors and the development of an improvement approach can be facilitators for learning together as well. It is important to not only measure the results of the network, but also to discuss these results in a multidisciplinary setting. A Dutch study showed that providing feedback and a benchmark improves the level of quality management in diabetes care groups as well [24]. Another recommendation is to involve patients in the development and monitoring of integrated care. The results show that different stakeholder groups experience the development of integrated care differently and that patients are barely involved in the development and monitoring of the diabetes care groups (one care group invited a patient representative for the survey). So, the inclusion of the patient’s perspective could be very valuable and helpful, especially on aspects related to patient-centeredness and quality of care. Internationally, more and more attention is given to larger roles for patients in the development of healthcare services as well [25]. The observation that the professional discipline or role in an integrated care service influences the assessment of the development of integrated care, reveals that the involved stakeholders have to be aware of the different perspectives of the involved stakeholder groups. Different stakeholder groups experience the development of their own care group differently. It is interesting that the players at a distance identify a lower percentage of present elements in their care group than the other two stakeholder groups. For the managers, coordinators and directors, it is a challenge to keep this group involved. It is important that all involved actors agree on what they want to achieve with each other and how they arrange their joint responsibility. It could be helpful to clearly discuss each other’s contributions and responsibilities and if the players at a distance should be involved in this process as well. From a patient’s perspective it is important that all stakeholders groups are well involved, because patients are dependent on the collective effort of these stakeholders.

As mentioned, the DMIC focuses on elements of integrated care defined as activities that can be undertaken, it does not measure delivered outcomes. For future research, it is interesting to relate the process aspects measured by the DMIC to the achieved outcomes. It is interesting to relate this results to client outcomes, for instance to how satisfied their patients are. Is there a relationship between these outcomes and the scores on the DMIC? In diabetes care there is a growing body of knowledge about which outcomes can be measured, so there are possibilities to relate these outcome measures with process measures. Another suggestion for further research is to carry out the self-assessment again to compare the results over time and to look at possible similarities and differences over time. It also could be interesting to study what the care groups did with the advices and reports of the researchers. Did they implement these advices in their care groups? In this light, the self-assessment tool and its results could be used as a starting point for care group discussions about development.

## Conclusions

Since the introduction of bundled payments in 2010, many care groups were initiated in the Netherlands. This led to more than 100 active care groups nowadays. In this light, it is relevant to systematically study the development of care groups. In this study the self-assessment monitoring of 36 different diabetes care groups is presented and compared. A first conclusion of this study is that the professionals in the diabetes care groups work together in multi-disciplinary teams with clear roles and tasks. However, the care groups can develop further on aspects related to performance management, the quality and the patient-centeredness of their services. This shows the complexity of implementing integrated care in practice and confirms the practical relevance of using a conceptual model like the DMIC. The last conclusion that can be drawn is that there is a substantial difference between stakeholder groups in the assessment of diabetes care. This suggests that the roles of stakeholders in integrated care services could influence the way they asses the development of integrated care, or that certain stakeholder groups could be less involved or informed. Anyhow, patients are dependent on the collective effort of the stakeholders. So, from a patient’s perspective, it is important to keep the stakeholders well involved and informed.

## Additional file

Additional file 1: Annex 1 Development Model for Integrated Care Questionnaire. Data collection instrument - blank, English copy of the questionnaire. (DOCX 31 kb)

## Abbreviations

DMIC: Development model of integrated care
M: Mean
SD: Standard deviation
