# Supplementary material for: The development of integrated diabetes care in the Netherlands: a multiplayer self-assessment analysis
Source: BMC Health Serv Res. 2017 Mar 21;17:219. doi: 10.1186/s12913-017-2167-6 (PMC5359897; doi:10.1186/s12913-017-2167-6)
Supplement: Additional file 1: — Annex 1 Development Model for Integrated Care Questionnaire. Data collection instrument - blank, English copy of the questionnaire. (DOCX 31 kb) [file 12913_2017_2167_MOESM1_ESM.docx]

**Annex 1: Development Model for Integrated Care Questionnaire**

**Part A: General information about the care group**

| **No.** | **Question:** | **Answer categories:** |
| --- | --- | --- |
| 1 | What is the name of the care group? | [text] |
| 2 | What year is the starting year of the collaboration in the care group? | [text] |
| 3a | Number of included clients in the year: | [total number of clients of last year] |
| 3b | Present care providers: | Tick box (multiple answers possible):   1. General practitioners 2. Hospital 3. Rehabilitation clinics 4. Nursing homes 5. Homes for the elderly 6. Home care organizations 7. Mental health care 8. Welfare organizations 9. Municipalities 10. Other: (ADD SPECIFIC SERVICES PER TYPE) |
| 3c | The total number of involved health care organizations | [text] |
| 3d | Are there collaboration agreements with the general practitioners? | 1. Yes 2. No |
| 3e | Are there collaboration agreements with ambulatory care services? | 1. Yes 2. No |
| 3f | Are there periodically meetings regarding this integrated care service with: | Tick box (multiple answers possible):   1. Health care insurers 2. Assessment agencies 3. Municipalities 4. Client federations 5. None of these |
| 4a | Are there working groups on an operational level which focus on the improvement and development of the integrated care service? | 1. Yes 2. No |
| 4a* | If yes: Who participates in these working groups? | [text] |
| 4b | Is there a coordinator/project/program leader on the level of the integrated care service which has coordinating tasks? | 1. Yes 2. No |
| 4b* | If yes: How many hours per week does this person have for these tasks? | [text - in hours per week] |
| 4c | Is there a formal collaboration agreement signed up by the CEO’s of the involved health care organizations? | 1. Yes 2. No |
| 4d | Are there regular meetings on CEO level/a steering committee regarding this integrated care service? | 1. Yes 2. No |

**Part B: Relevance and presence of the elements per cluster**

| **No.** | **No. in cluster** | **Cluster:** | **Element:** | **1. Is this element relevant?** | **2. Is this element present in the care group?** | **3. Does this element have priority to be implemented?** |
| --- | --- | --- | --- | --- | --- | --- |
| 1 | 1 | Patient centeredness | Developing a front office: single entry point for client information | a. Yes (proceed to question 2) b. No | a. Yes  b. No (proceed to question 3) c. No judgement | a. Yes  b. No c. No judgement |
| 2 | 2 | Patient centeredness | Collaboratively offering client information of the care partners | a. Yes (proceed to question 2) b. No | a. Yes  b. No (proceed to question 3) c. No judgement | a. Yes  b. No c. No judgement |
| 3 | 3 | Patient centeredness | Providing understandable and client-centered information | a. Yes (proceed to question 2) b. No | a. Yes  b. No (proceed to question 3) c. No judgement | a. Yes  b. No c. No judgement |
| 4 | 4 | Patient centeredness | Using self-management support methods as a part of integrated care | a. Yes (proceed to question 2) b. No | a. Yes  b. No (proceed to question 3) c. No judgement | a. Yes  b. No c. No judgement |
| 5 | 5 | Patient centeredness | Implementing care process-supporting clinical information systems | a. Yes (proceed to question 2) b. No | a. Yes  b. No (proceed to question 3) c. No judgement | a. Yes  b. No c. No judgement |
| 6 | 6 | Patient centeredness | Developing care programmes for relevant client subgroups | a. Yes (proceed to question 2) b. No | a. Yes  b. No (proceed to question 3) c. No judgement | a. Yes  b. No c. No judgement |
| 7 | 7 | Patient centeredness | Flexible adjustment of integrated care corresponding to individual clients’ needs | a. Yes (proceed to question 2) b. No | a. Yes  b. No (proceed to question 3) c. No judgement | a. Yes  b. No c. No judgement |
| 8 | 8 | Patient centeredness | Designing care for clients with multi- or co-morbidities | a. Yes (proceed to question 2) b. No | a. Yes  b. No (proceed to question 3) c. No judgement | a. Yes  b. No c. No judgement |
| 9 | 9 | Patient centeredness | Using a protocol for the systematic follow-up of clients | a. Yes (proceed to question 2) b. No | a. Yes  b. No (proceed to question 3) c. No judgement | a. Yes  b. No c. No judgement |
| 10 | 1 | Delivery system | Reaching agreements on logistics (e.g. waiting periods and throughput time | a. Yes (proceed to question 2) b. No | a. Yes  b. No (proceed to question 3) c. No judgement | a. Yes  b. No c. No judgement |
| 11 | 2 | Delivery system | Developing criteria for the inclusion and throughput of clients in the care service | a. Yes (proceed to question 2) b. No | a. Yes  b. No (proceed to question 3) c. No judgement | a. Yes  b. No c. No judgement |
| 12 | 3 | Delivery system | Reaching agreements on referrals and the transfer of clients through the care service | a. Yes (proceed to question 2) b. No | a. Yes  b. No (proceed to question 3) c. No judgement | a. Yes  b. No c. No judgement |
| 13 | 4 | Delivery system | Reaching agreements on linking clients to outside resources or community care partners | a. Yes (proceed to question 2) b. No | a. Yes  b. No (proceed to question 3) c. No judgement | a. Yes  b. No c. No judgement |
| 14 | 5 | Delivery system | Reaching agreements among care partners on scheduling client examinations and treatment | a. Yes (proceed to question 2) b. No | a. Yes  b. No (proceed to question 3) c. No judgement | a. Yes  b. No c. No judgement |
| 15 | 6 | Delivery system | Developing criteria for assessing clients’ urgency | a. Yes (proceed to question 2) b. No | a. Yes  b. No (proceed to question 3) c. No judgement | a. Yes  b. No c. No judgement |
| 16 | 7 | Delivery system | Reaching agreements among care partners on discharge planning | a. Yes (proceed to question 2) b. No | a. Yes  b. No (proceed to question 3) c. No judgement | a. Yes  b. No c. No judgement |
| 17 | 8 | Delivery system | Reaching agreements among care partners on managing client preferences | a. Yes (proceed to question 2) b. No | a. Yes  b. No (proceed to question 3) c. No judgement | a. Yes  b. No c. No judgement |
| 18 | 9 | Delivery system | Reaching agreements among care partners on providing care to waiting-list clients | a. Yes (proceed to question 2) b. No | a. Yes  b. No (proceed to question 3) c. No judgement | a. Yes  b. No c. No judgement |
| 19 | 10 | Delivery system | Reaching agreements on procedures for information exchange | a. Yes (proceed to question 2) b. No | a. Yes  b. No (proceed to question 3) c. No judgement | a. Yes  b. No c. No judgement |
| 20 | 11 | Delivery system | Reaching agreements on procedures for the exchange of client information | a. Yes (proceed to question 2) b. No | a. Yes  b. No (proceed to question 3) c. No judgement | a. Yes  b. No c. No judgement |
| 21 | 12 | Delivery system | Using uniform client-identification numbers within the care service | a. Yes (proceed to question 2) b. No | a. Yes  b. No (proceed to question 3) c. No judgement | a. Yes  b. No c. No judgement |
| 22 | 13 | Delivery system | Developing connections with the databases of partners in the care service | a. Yes (proceed to question 2) b. No | a. Yes  b. No (proceed to question 3) c. No judgement | a. Yes  b. No c. No judgement |
| 23 | 14 | Delivery system | Using a single client-monitoring record accessible to all care partners | a. Yes (proceed to question 2) b. No | a. Yes  b. No (proceed to question 3) c. No judgement | a. Yes  b. No c. No judgement |
| 24 | 15 | Delivery system | Using shared client treatment and care plans | a. Yes (proceed to question 2) b. No | a. Yes  b. No (proceed to question 3) c. No judgement | a. Yes  b. No c. No judgement |
| 25 | 16 | Delivery system | Reaching agreements among care partners on the consultation of experts and professionals | a. Yes (proceed to question 2) b. No | a. Yes  b. No (proceed to question 3) c. No judgement | a. Yes  b. No c. No judgement |
| 26 | 17 | Delivery system | Deploying specialized nurses within the care service | a. Yes (proceed to question 2) b. No | a. Yes  b. No (proceed to question 3) c. No judgement | a. Yes  b. No c. No judgement |
| 27 | 18 | Delivery system | Offering case management to clients with complex needs | a. Yes (proceed to question 2) b. No | a. Yes  b. No (proceed to question 3) c. No judgement | a. Yes  b. No c. No judgement |
| 28 | 1 | Performance management | Defining performance indicators to evaluate the results of the integrated care delivered | a. Yes (proceed to question 2) b. No | a. Yes  b. No (proceed to question 3) c. No judgement | a. Yes  b. No c. No judgement |
| 29 | 2 | Performance management | Establishing quality targets for the performance of the whole care service | a. Yes (proceed to question 2) b. No | a. Yes  b. No (proceed to question 3) c. No judgement | a. Yes  b. No c. No judgement |
| 30 | 3 | Performance management | Establishing quality targets for the performance of care partners | a. Yes (proceed to question 2) b. No | a. Yes  b. No (proceed to question 3) c. No judgement | a. Yes  b. No c. No judgement |
| 31 | 4 | Performance management | Gathering data on client logistics (e.g. volumes, waiting periods and throughput times) in the care service | a. Yes (proceed to question 2) b. No | a. Yes  b. No (proceed to question 3) c. No judgement | a. Yes  b. No c. No judgement |
| 32 | 5 | Performance management | Gathering client-related performance data (health status, quality of life) | a. Yes (proceed to question 2) b. No | a. Yes  b. No (proceed to question 3) c. No judgement | a. Yes  b. No c. No judgement |
| 33 | 6 | Performance management | Monitoring client judgements and satisfaction for the whole care service | a. Yes (proceed to question 2) b. No | a. Yes  b. No (proceed to question 3) c. No judgement | a. Yes  b. No c. No judgement |
| 34 | 7 | Performance management | Gathering financial performance data of the care service | a. Yes (proceed to question 2) b. No | a. Yes  b. No (proceed to question 3) c. No judgement | a. Yes  b. No c. No judgement |
| 35 | 8 | Performance management | Making transparent the effects of the collaboration on the production of the care partners | a. Yes (proceed to question 2) b. No | a. Yes  b. No (proceed to question 3) c. No judgement | a. Yes  b. No c. No judgement |
| 36 | 9 | Performance management | Reaching agreements about the uniform use of performance indicators in the care service | a. Yes (proceed to question 2) b. No | a. Yes  b. No (proceed to question 3) c. No judgement | a. Yes  b. No c. No judgement |
| 37 | 10 | Performance management | Using a systematic procedure for the evaluation of agreements, approaches and results | a. Yes (proceed to question 2) b. No | a. Yes  b. No (proceed to question 3) c. No judgement | a. Yes  b. No c. No judgement |
| 38 | 11 | Performance management | Monitoring and analyzing mistakes/near mistakes in the care service | a. Yes (proceed to question 2) b. No | a. Yes  b. No (proceed to question 3) c. No judgement | a. Yes  b. No c. No judgement |
| 39 | 12 | Performance management | Monitoring successes and results during the development of the integrated care service | a. Yes (proceed to question 2) b. No | a. Yes  b. No (proceed to question 3) c. No judgement | a. Yes  b. No c. No judgement |
| 40 | 13 | Performance management | Installing improvement teams at the care-chain level | a. Yes (proceed to question 2) b. No | a. Yes  b. No (proceed to question 3) c. No judgement | a. Yes  b. No c. No judgement |
| 41 | 14 | Performance management | Providing feedback to care partners on transfers | a. Yes (proceed to question 2) b. No | a. Yes  b. No (proceed to question 3) c. No judgement | a. Yes  b. No c. No judgement |
| 42 | 15 | Performance management | Using feedback and reminders by professionals for improving care | a. Yes (proceed to question 2) b. No | a. Yes  b. No (proceed to question 3) c. No judgement | a. Yes  b. No c. No judgement |
| 43 | 16 | Performance management | Using evidence-based guidelines and standards | a. Yes (proceed to question 2) b. No | a. Yes  b. No (proceed to question 3) c. No judgement | a. Yes  b. No c. No judgement |
| 44 | 1 | Quality care | Systematically assessing the needs of the clients in the care service | a. Yes (proceed to question 2) b. No | a. Yes  b. No (proceed to question 3) c. No judgement | a. Yes  b. No c. No judgement |
| 45 | 2 | Quality care | Developing a multidisciplinary care pathway | a. Yes (proceed to question 2) b. No | a. Yes  b. No (proceed to question 3) c. No judgement | a. Yes  b. No c. No judgement |
| 46 | 3 | Quality care | Monitoring whether the care delivered corresponds with the evidence-based guidelines | a. Yes (proceed to question 2) b. No | a. Yes  b. No (proceed to question 3) c. No judgement | a. Yes  b. No c. No judgement |
| 47 | 4 | Quality care | Involving client representatives in monitoring the performance of the care service | a. Yes (proceed to question 2) b. No | a. Yes  b. No (proceed to question 3) c. No judgement | a. Yes  b. No c. No judgement |
| 48 | 5 | Quality care | Involving client representatives in improvement projects in the care service | a. Yes (proceed to question 2) b. No | a. Yes  b. No (proceed to question 3) c. No judgement | a. Yes  b. No c. No judgement |
| 49 | 1 | Result-focused learning | Defining and assessing the characteristics of the collaboratively delivered care | a. Yes (proceed to question 2) b. No | a. Yes  b. No (proceed to question 3) c. No judgement | a. Yes  b. No c. No judgement |
| 50 | 2 | Result-focused learning | Making the benefits of the collaboration transparent for each care-chain partner | a. Yes (proceed to question 2) b. No | a. Yes  b. No (proceed to question 3) c. No judgement | a. Yes  b. No c. No judgement |
| 51 | 3 | Result-focused learning | Linking consequences to the achievement of goals agreed upon | a. Yes (proceed to question 2) b. No | a. Yes  b. No (proceed to question 3) c. No judgement | a. Yes  b. No c. No judgement |
| 52 | 4 | Result-focused learning | Integrating incentives for rewarding the achievement of quality targets | a. Yes (proceed to question 2) b. No | a. Yes  b. No (proceed to question 3) c. No judgement | a. Yes  b. No c. No judgement |
| 53 | 5 | Result-focused learning | Using knowledge and information for directing and coordinating the care service | a. Yes (proceed to question 2) b. No | a. Yes  b. No (proceed to question 3) c. No judgement | a. Yes  b. No c. No judgement |
| 54 | 6 | Result-focused learning | Sharing knowledge among care partners about effectively organizing sustainable integrated care | a. Yes (proceed to question 2) b. No | a. Yes  b. No (proceed to question 3) c. No judgement | a. Yes  b. No c. No judgement |
| 55 | 7 | Result-focused learning | Collaboratively assessing bottlenecks and gaps in care | a. Yes (proceed to question 2) b. No | a. Yes  b. No (proceed to question 3) c. No judgement | a. Yes  b. No c. No judgement |
| 56 | 8 | Result-focused learning | Collaborative learning in the care service in order to innovate integrated care | a. Yes (proceed to question 2) b. No | a. Yes  b. No (proceed to question 3) c. No judgement | a. Yes  b. No c. No judgement |
| 57 | 9 | Result-focused learning | Striving toward an open culture for discussing possible improvements for care partners | a. Yes (proceed to question 2) b. No | a. Yes  b. No (proceed to question 3) c. No judgement | a. Yes  b. No c. No judgement |
| 58 | 10 | Result-focused learning | Stimulating a learning culture and continuous improvement in the care service | a. Yes (proceed to question 2) b. No | a. Yes  b. No (proceed to question 3) c. No judgement | a. Yes  b. No c. No judgement |
| 59 | 11 | Result-focused learning | Learning by the exchange of information among professionals about the care process | a. Yes (proceed to question 2) b. No | a. Yes  b. No (proceed to question 3) c. No judgement | a. Yes  b. No c. No judgement |
| 60 | 12 | Result-focused learning | Introducing collaborative education programmes and learning environments for the care professionals | a. Yes (proceed to question 2) b. No | a. Yes  b. No (proceed to question 3) c. No judgement | a. Yes  b. No c. No judgement |
| 61 | 1 | Inter-professional teamwork | Defining the targeted client group | a. Yes (proceed to question 2) b. No | a. Yes  b. No (proceed to question 3) c. No judgement | a. Yes  b. No c. No judgement |
| 62 | 2 | Inter-professional teamwork | Working in multidisciplinary teams | a. Yes (proceed to question 2) b. No | a. Yes  b. No (proceed to question 3) c. No judgement | a. Yes  b. No c. No judgement |
| 63 | 3 | Inter-professional teamwork | Reaching agreements on the availability and accessibility of professionals | a. Yes (proceed to question 2) b. No | a. Yes  b. No (proceed to question 3) c. No judgement | a. Yes  b. No c. No judgement |
| 64 | 1 | Roles and tasks | Directing the care service by appointing a limited number of people with coordinating tasks | a. Yes (proceed to question 2) b. No | a. Yes  b. No (proceed to question 3) c. No judgement | a. Yes  b. No c. No judgement |
| 65 | 2 | Roles and tasks | Installing a coordinator working at the chain-care level | a. Yes (proceed to question 2) b. No | a. Yes  b. No (proceed to question 3) c. No judgement | a. Yes  b. No c. No judgement |
| 66 | 3 | Roles and tasks | Reaching agreements among care partners on tasks, responsibilities and authorizations | a. Yes (proceed to question 2) b. No | a. Yes  b. No (proceed to question 3) c. No judgement | a. Yes  b. No c. No judgement |
| 67 | 4 | Roles and tasks | Establishing the roles and tasks of multidisciplinary team members | a. Yes (proceed to question 2) b. No | a. Yes  b. No (proceed to question 3) c. No judgement | a. Yes  b. No c. No judgement |
| 68 | 5 | Roles and tasks | Ensuring that professionals in the care service are informed of one another’s expertise and tasks | a. Yes (proceed to question 2) b. No | a. Yes  b. No (proceed to question 3) c. No judgement | a. Yes  b. No c. No judgement |
| 69 | 6 | Roles and tasks | Achieving adjustments among care partners by means of direct contact | a. Yes (proceed to question 2) b. No | a. Yes  b. No (proceed to question 3) c. No judgement | a. Yes  b. No c. No judgement |
| 70 | 7 | Roles and tasks | Realizing direct contact among professionals in the care service | a. Yes (proceed to question 2) b. No | a. Yes  b. No (proceed to question 3) c. No judgement | a. Yes  b. No c. No judgement |
| 71 | 8 | Roles and tasks | Reaching agreements on introducing and integrating new partners in the care service | a. Yes (proceed to question 2) b. No | a. Yes  b. No (proceed to question 3) c. No judgement | a. Yes  b. No c. No judgement |
| 72 | 1 | Commitment | Defining the ambitions and aims of the collaboration in the care service | a. Yes (proceed to question 2) b. No | a. Yes  b. No (proceed to question 3) c. No judgement | a. Yes  b. No c. No judgement |
| 73 | 2 | Commitment | Signing collaboration agreements among the care partners | a. Yes (proceed to question 2) b. No | a. Yes  b. No (proceed to question 3) c. No judgement | a. Yes  b. No c. No judgement |
| 74 | 3 | Commitment | Establishing dependencies among care partners | a. Yes (proceed to question 2) b. No | a. Yes  b. No (proceed to question 3) c. No judgement | a. Yes  b. No c. No judgement |
| 75 | 4 | Commitment | Guiding the care service by emphasizing a collaborative commitment | a. Yes (proceed to question 2) b. No | a. Yes  b. No (proceed to question 3) c. No judgement | a. Yes  b. No c. No judgement |
| 76 | 5 | Commitment | Assuring the leadership commitment of the partners involved in the care service | a. Yes (proceed to question 2) b. No | a. Yes  b. No (proceed to question 3) c. No judgement | a. Yes  b. No c. No judgement |
| 77 | 6 | Commitment | Stimulating the awareness of working in a care service | a. Yes (proceed to question 2) b. No | a. Yes  b. No (proceed to question 3) c. No judgement | a. Yes  b. No c. No judgement |
| 78 | 7 | Commitment | Structural meetings of the leaders of the care-chain organizations | a. Yes (proceed to question 2) b. No | a. Yes  b. No (proceed to question 3) c. No judgement | a. Yes  b. No c. No judgement |
| 79 | 8 | Commitment | Structural meetings with external parties, such as insurers, local governments and inspectorates | a. Yes (proceed to question 2) b. No | a. Yes  b. No (proceed to question 3) c. No judgement | a. Yes  b. No c. No judgement |
| 80 | 9 | Commitment | Describing the tasks and authorities of leaders, coordinators and advisory boards in the care service | a. Yes (proceed to question 2) b. No | a. Yes  b. No (proceed to question 3) c. No judgement | a. Yes  b. No c. No judgement |
| 81 | 10 | Commitment | Stimulating trust among care partners | a. Yes (proceed to question 2) b. No | a. Yes  b. No (proceed to question 3) c. No judgement | a. Yes  b. No c. No judgement |
| 82 | 11 | Commitment | Reaching agreements about letting go care partner domains | a. Yes (proceed to question 2) b. No | a. Yes  b. No (proceed to question 3) c. No judgement | a. Yes  b. No c. No judgement |
| 83 | 1 | Transparent entrepreneurship | Making a commitment to a joint responsibility for the final goals and results to be achieved | a. Yes (proceed to question 2) b. No | a. Yes  b. No (proceed to question 3) c. No judgement | a. Yes  b. No c. No judgement |
| 84 | 2 | Transparent entrepreneurship | Reaching agreements on the financial budget for integrated care | a. Yes (proceed to question 2) b. No | a. Yes  b. No (proceed to question 3) c. No judgement | a. Yes  b. No c. No judgement |
| 85 | 3 | Transparent entrepreneurship | Allocating financial budgets for the implementation and maintenance of integrated care | a. Yes (proceed to question 2) b. No | a. Yes  b. No (proceed to question 3) c. No judgement | a. Yes  b. No c. No judgement |
| 86 | 4 | Transparent entrepreneurship | Offering a single collaborative financial contract to the financing parties through the collective of care partners | a. Yes (proceed to question 2) b. No | a. Yes  b. No (proceed to question 3) c. No judgement | a. Yes  b. No c. No judgement |
| 87 | 5 | Transparent entrepreneurship | Creating an open environment that encourages experiments and pilot projects | a. Yes (proceed to question 2) b. No | a. Yes  b. No (proceed to question 3) c. No judgement | a. Yes  b. No c. No judgement |
| 88 | 6 | Transparent entrepreneurship | Involving leaders in improvement efforts in the care service | a. Yes (proceed to question 2) b. No | a. Yes  b. No (proceed to question 3) c. No judgement | a. Yes  b. No c. No judgement |
| 89 | 7 | Transparent entrepreneurship | Using a uniform language in the care service | a. Yes (proceed to question 2) b. No | a. Yes  b. No (proceed to question 3) c. No judgement | a. Yes  b. No c. No judgement |

**Part C: Phases of development**
The DMIC describes four possible phases of development of integrated care. In this part the question is in which phase of development your integrated care service is best recognized. After you assessed the phase of development, some questions about this phases and previous phases are being addressed.

First, please read the text of the four development phases:

*PHASE 1 Initiative and design phase:*

The collaboration between health care providers has been intensified or started up. The starting point is a common problem or chance occurrence, or builds on current cooperation among care professionals. There is a sense of urgency and there are possibilities for working on these challenges in collaboration. The targeted patient group, the care chain and care process have been defined, as also the needs of patients and stakeholders. The level of ambitions, motivation and leadership determine the progress achieved. A multidisciplinary team designs an experiment or project to execute the current ideas. The collaboration can be signed up to in an agreement among care partners.

*Key words*: Exploring possibilities/impossibilities, ambitions and chances, (project) design and collaboration agreements.

*PHASE 2 Experimental and execution phase:*

New initiatives or projects are being executed in the care chain. The aims, content, roles, and tasks in the care chain have been clarified and written down in care pathways and protocols. There is coordination at the level of the care chain by for instance installing coordinators or setting up meetings. Information about patient groups, working procedures or professional knowledge is exchanged. There are experiments within the collaboration, results are evaluated to learn from and reflect on. Preconditions for projects have been considered and boundary conditions have been solved by collaborative means or agreements among care providers.

*Key words*: Writing down aims and content of the collaboration, coordination at care chain level, experimenting and reflecting.

*PHASE 3 Expansion and monitoring phase:*

Projects have been expanded or integrated in integrated care programmes. Agreements on the content, tasks and roles within the care chain are clear and signed up. Collaboration is no longer on an informal basis. Results are systematically monitored and improvement areas identified. The targeted population has been surveyed. More collaborative initiatives emerge such as mutual education programmes. There is a continuous commitment to the ambition of the integrated care programme. Interorganizational barriers and fragmented financial structures are on the agenda of the care partners. *Key words*: Further development and maturity, monitoring and improving results, new questions and innovation.

*PHASE 4 Consolidation and transformation phase:*

The integrated care programme is the regular way of working and providing care. Coordination at care chain level is operational; information is shared, transferred and fed back. A monitoring system periodically shows if results are being sustained, what specific improvement possibilities have been identified and to what extent patient needs have been met. The programme builds further on successful results. Organizational structures transform or are newly designed around the integrated care programme. Financial agreements are arranged with financers by means of integral contracts covering the care chain as a whole. Partners in the care chain explore new options for collaboration in the external environment with other partners.

*Key words*: Continuous improvement, new ambitions, structures fitting the integrated care programme (organizational structures, integral financing).

| **No.** | **Question:** | **Answer categories:** |
| --- | --- | --- |
| 1a | Which phase description characterizes your integrated care service at this moment best? | 1. Phase 1, the initiative and design phase 2. Phase 2, the experimental and execution phase 3. Phase 3, the expansion and monitoring phase 4. Phase 4, the consolidation and transformation phase |
| 1b | Please explain your answer | [text] |
| 2 | Since when does your care group experience this phase? | [month, year] |
| 3 | According to you, how long will these phase take before entering the next phase? | [answer in months] |

The next questions are about your previous phase. Please read again the description of the previous phase of your answer of question C1a (IF QUESTION 1a’s ANSWER WAS phase 1, THIS QUESTION CAN BE SKIPPED)

| **No.** | **Question:** | **Answer categories:** |
| --- | --- | --- |
| 4a | Has your care group experienced this phase? | 1. Yes 2. No |
| 4b | Please explain your answer | [text] |
| 5 | How long took this phase? | [answer in months] |
| 6 | What was crucial for reaching the current phase, coming from the previous phase? | [text] |
